# The Effects of NAD^+^ on Apoptotic Neuronal Death and Mitochondrial Biogenesis and Function after Glutamate Excitotoxicity

**DOI:** 10.3390/ijms151120449

**Published:** 2014-11-07

**Authors:** Xiaowan Wang, Hailong Li, Shinghua Ding

**Affiliations:** Dalton Cardiovascular Research Center, Department of Bioengineering, University of Missouri, Columbia, MO 65211, USA; E-Mails: xw825@mail.missouri.edu (X.W.); hailongli0925@gmail.com (H.L.)

**Keywords:** ischemic stroke, glutamate excitotoxicity, apoptosis, AIF, mitochondrial biogenesis

## Abstract

NAD^+^ is an essential co-enzyme for cellular energy metabolism and is also involved as a substrate for many cellular enzymatic reactions. It has been shown that NAD^+^ has a beneficial effect on neuronal survival and brain injury in *in vitro* and *in vivo* ischemic models. However, the effect of NAD^+^ on mitochondrial biogenesis and function in ischemia has not been well investigated. In the present study, we used an *in vitro* glutamate excitotoxicity model of primary cultured cortical neurons to study the effect of NAD^+^ on apoptotic neuronal death and mitochondrial biogenesis and function. Our results show that supplementation of NAD^+^ could effectively reduce apoptotic neuronal death, and apoptotic inducing factor translocation after neurons were challenged with excitotoxic glutamate stimulation. Using different approaches including confocal imaging, mitochondrial DNA measurement and Western blot analysis of PGC-1 and NRF-1, we also found that NAD^+^ could significantly attenuate glutamate-induced mitochondrial fragmentation and the impairment of mitochondrial biogenesis. Furthermore, NAD^+^ treatment effectively inhibited mitochondrial membrane potential depolarization and NADH redistribution after excitotoxic glutamate stimulation. Taken together, our results demonstrated that NAD^+^ is capable of inhibiting apoptotic neuronal death after glutamate excitotoxicity via preserving mitochondrial biogenesis and integrity. Our findings provide insights into potential neuroprotective strategies in ischemic stroke.

## 1. Introduction

Cerebral ischemia, accounting for approximately 80% of all human strokes, results from the blockage of blood flow leading to the CNS and thus restricts delivery of glucose and oxygen, which are essential substrates for energy production, to the brain [1]. After ischemic stroke, neurons have different fates in the different regions. In the ischemic core, neurons die rapidly from the onset of ischemia due to glutamate and Ca^2+^ excitotoxicity resulting from severe energy depletion and the ensuing breakdown of ion homeostasis [2]. In the penumbra, the brain tissue is perfused with collateral blood flow and energy metabolism is partially preserved, thus the primary goal for stroke therapy is to salvage the penumbra in acute phase of ischemic stroke [1]. As the brain is an organ that needs high energy consumption and is especially sensitive to an ischemic insult, identifying a novel approach that can rescue or compensate for energy depletion after ischemia is important to minimize ischemia-induced neuronal death and brain injury.

NAD^+^ is an important co-enzyme for many enzymatic reactions in the tricarboxylic acid (TCA) cycle and oxidative phosphorylation in mitochondria, and glycolysis in cytoplasm. Thus NAD^+^ is required for the mitochondrial respiration and ATP synthesis and maintenance of NAD^+^ level is important for the prevention of mitochondrial dysfunction under the energy stress condition such as an ischemic insult [3]. NAD^+^ is also involved in a number of signaling pathways in mammalian cells. NAD^+^ is a substrate of poly(ADP-ribose) polymerase 1 (PARP-1) [4] and a mammalian family of deacetylase called sirtuins (SIRT1-7) [5,6,7,8]. NAD^+^ is also highly correlated with mitochondrial biogenesis [9,10,11]. Our and other studies have shown that supplementation of NAD^+^ can exert a neuronal protective effect in *in vitro* and *in vivo* ischemic models, and maintaining intracellular NAD^+^ levels is important in promoting cell survival during ischemia [12,13,14]. It was also reported that ischemia causes the reduction of NAD^+^ levels [13,15,16]. These studies provide solid evidence that NAD^+^ can protect neurons from death following ischemia. However, the mechanism of NAD^+^ protective effect on cerebral ischemia in the context of mitochondrial dysfunction has not been well investigated.

In the present study, we used an *in vitro* glutamate excitotoxicity model of primary cultured cortical neurons, which can mimic the penumbra in focal ischemic stroke, to study the effect of NAD^+^ on apoptotic neuronal death, AIF translocation, mitochondrial biogenesis and function. Using terminal dinucleotidyltransferase-mediated UTP end labeling (TUNEL) and immunostaining, we studied the effect of exogenous NAD^+^ on apoptotic neuronal death and apoptotic inducing factor (AIF) translocation from mitochondria to nucleus after excitotoxic glutamate stimulation. Using fluorescent imaging, quantitative PCR (qPCR) and Western blot analysis, we further investigated the effect of NAD^+^ on mitochondrial fragmentation and the impairment of mitochondrial biogenesis after glutamate excitotoxicity by measuring mitochondrial DNA (mtDNA), proliferator-activated receptor γ coactivator 1α (PGC-1), and nuclear respiratory factor (NRF-1) levels in neurons. In addition, we also studied the effect of NAD^+^ treatment on mitochondrial membrane potential (MMP) depolarization induced by glutamate stimulation. Thus, NAD^+^ is capable of promoting neuronal survival after glutamate excitotoxicity via preserving mitochondrial integrity and biogenesis. Our results provide insights into potential strategies of ameliorating ischemia-induced neuronal death and brain injury.

## 2. Results

### 2.1. NAD^+^ Ameliorates Apoptotic Neuronal Death after Glutamate Stimulation

**Figure 1:**
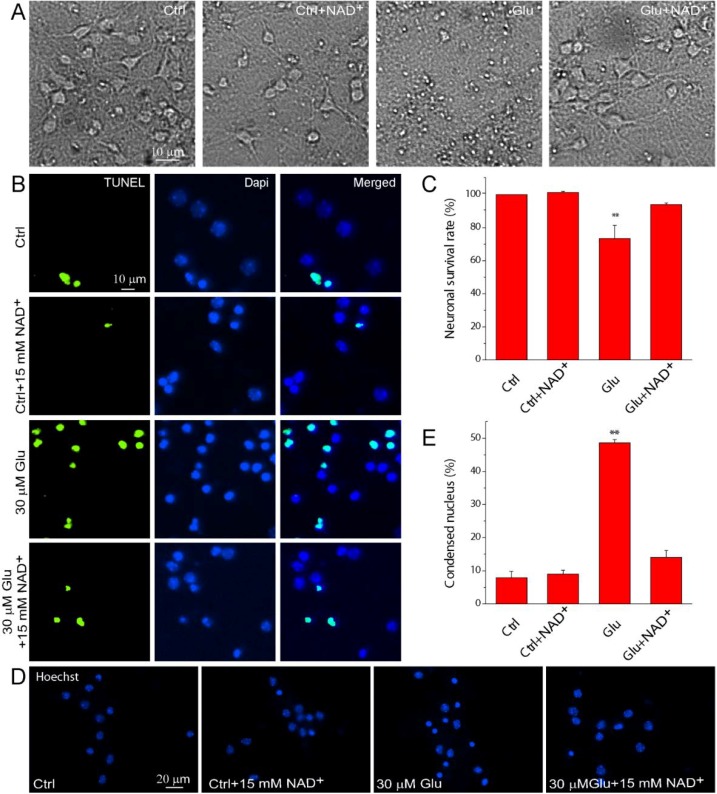
Exogenous NAD^+^ supplementation ameliorates apoptosis in primary mouse neuronal cultures after glutamate stimulation. (**A**) Phase contrast images of mouse cortical neurons without and with treatment of 30 µM glutamate together with 3 μM glycine for 24 h in the presence and absence of 15 mM NAD^+^; (**B**) Representative fluorescent images showing the double staining of TUNEL and DNA-binding dye DAPI in neurons. Neuronal cultures were treated with glutamate and NAD^+^ as in (**A**); (**C**) Neuronal survival rates under different conditions; (**D**) Hoechst 33342 nuclear staining in control neurons and neurons exposed to 30 μM glutamate together with 3 μM glycine in the presence and absence of 15 mM NAD^+^ for 24 h; (**E**) Percentages of condensed nuclei based on Hoechst 33342 staining were quantified in each condition. Data are shown as mean ± SE; *n* = 3 coverslips per experimental condition from 3 independent experiments. ** *p* < 0.01 *versus* Ctrl, Ctrl + NAD^+^ and Glu + NAD^+^, ANOVA test.

We initially investigated the effect of exogenous NAD^+^ on apoptotic neuronal death after glutamate excitotoxicity in primary mouse cortical neuronal cultures. Representative images show that stimulation of neurons with 30 μM glutamate together with 3 μM glycine for 24 h resulted in the condensation of neuronal soma (Figure 1A) while the addition of 15 mM NAD^+^ in neuronal cultures maintained normal neuronal morphology after glutamate excitotoxicity. Apoptosis was evaluated using TUNEL and Hoechst 33342 stainings. TUNEL+ neurons have condensed, shrunken and fragmented nuclei (Figure 1B). Our results show that glutamate treatment lead to apoptosis in large amount of neurons; however, supplementation of 15 mM NAD^+^ in neurons significantly reduced the number of TUNEL+ neurons and increased neuronal survival rate (Figure 1C). The results were further confirmed by Hoechst 33342 staining. NAD^+^ can significantly reduce the number of neurons with condensed nucleus (Figure 1D) after glutamate stimulation. Quantitative results show that glutamate treatment lead to condensed nucleus in 50% of neurons and NAD^+^ decreased this number close to the control level (Figure 1E). Thus, using two independent assays, our results demonstrated that exogenous supplementation of NAD^+^ can protect neurons against glutamate-induced apoptosis.

### 2.2. NAD^+^ Prevents the Translocation of AIF from Mitochondria to Nucleus

**Figure 2:**
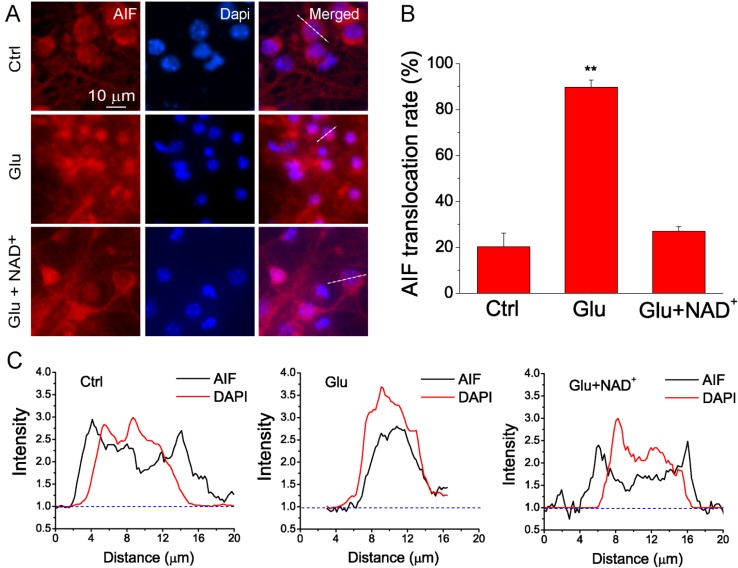
NAD^+^ prevents the translocation of apoptotic inducing factor (AIF) from mitochondria to nucleus after glutamate excitotoxicity. (**A**) Representative images of primary neuronal cultures stained with AIF antibody. Neurons were treated with 100 μM glutamate together with 10 μM glycine in the absence and presence of 15 mM NAD^+^ for 3 h. Nuclei were counterstained with DAPI. Translocation of AIF from mitochondria to nuclei is illustrated by the overlap of AIF (red) and DAPI (blue) staining; (**B**) Summary of AIF translocation after glutamate stimulation. Data was quantified by the number of cells with overlap of AIF and DAPI among the total number of cells determined by DAPI staining. Data are shown as mean ± SE; *n* = 2–3 coverslips per experimental condition from three independent experiments. ** *p* < 0.01 *versus* Ctrl and Glu + NAD^+^, ANOVA test; (**C**) Line-scan of AIF and DAPI fluorescence from the cells indicated in (**A**). The fluorescence was normalized to the background. Notice the translocation of AIF after glutamate stimulation and prevention of AIF translocation by NAD^+^.

It has been known that ischemia leads to the translocation of AIF from mitochondria to nucleus [17,18]. Accumulation of AIF in the nucleus will induce chromatin condensation, large-scale DNA fragmentation and eventually caspase-independent apoptotic cell death [19]. To investigate the neuroprotective mechanism of NAD^+^ in glutamate excitotoxicity, we studied whether NAD^+^ can inhibit the translocation of AIF from mitochondria to nucleus after glutamate stimulation. Neurons were exposed to 100 μM glutamate together with 10 μM glycine for 3 h and immunostaining of AIF was then conducted. The effect of NAD^+^ on the AIF translocation was evaluated using fluorescent imaging. Our data show that AIF was largely located in cytoplasm under control conditions, while glutamate stimulation led to the concentration of AIF in the nuclei of neurons. Treatment of neurons with 15 mM NAD^+^ significantly prevented glutamate-induced AIF translocation to nucleus (Figure 2A). Quantitative analysis show that about 90% of neurons exhibited AIF translocation after glutamate treatment, while only about 20% of neurons have concentrated AIF in the nuclei under control conditions. Supplementation of NAD^+^ can reduce the percentage of neurons with AIF translocation close to the control levels (Figure 2B). These results demonstrate that NAD^+^ can prevent the translocation of AIF from mitochondria to nucleus after glutamate excitotoxicity suggesting NAD^+^ can reduce caspase-independent apoptosis. Furthermore, line-scan analysis of AIF and DAPI fluorescence clearly shows that glutamate stimulation induced translocation of AIF from cytosol to nuclei, but NAD^+^ could reverse this process (Figure 2C).

### 2.3. NAD^+^ Inhibits the Mitochondrial Fragmentation after Glutamate Excitotoxicity

**Figure 3:**
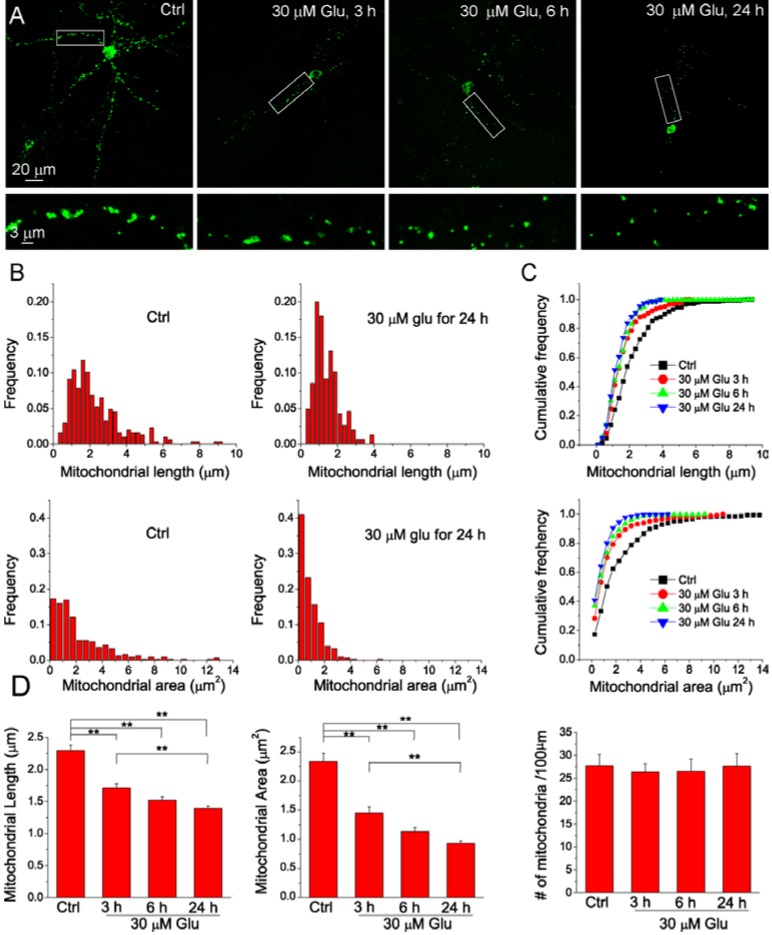
The time course of mitochondrial fragmentation after glutamate stimulation. (**A**) Maximal projection confocal images of primary cortical neurons transfected with mito-AcGFP1. Neurons were treated without or with 30 μM glutamate together with 3 μM glycine for 3, 6 and 24 h, respectively. The lower panels are the high resolution images of the boxed region in upper panels. The images at different time points were acquired from different neurons; (**B**–**C**) Frequency distribution and cumulative curves of mitochondrial length and area under different conditions. The values of histogram intervals (bins) are 0.25 μm for mitochondrial length and 0.5 μm^2^ for mitochondrial area. Distribution data showed that glutamate treatment caused significant increase in the number of shorter and smaller mitochondria and this effect was dependent on treatment time; (**D**) The summary of average mitochondrial length, area and density for each condition. Data are shown as mean ± SE; ** *p* < 0.01, ANOVA test. Data were collected from 10–11 neurons grown on two glass coverslip per experimental condition and a total of 1169 mitochondria were measured.

Mitochondria are highly dynamic organelles with the length, size and shape controlled by fission and fusion [20,21]. The balance of fission and fusion is disrupted in neuronal injury and degeneration, thus causing morphological change and structural damage associated with mitochondrial dysfunction [22,23,24]. Mitochondrial fragmentation has been widely detected after apoptosis [25]. Here, we initially studied the time course of mitochondrial fragmentation after glutamate stimulation. Neurons were transfected with mitochondria-targeted AcGFP (mito-AcGFP) to visualize the morphology of mitochondria. Individual mitochondrion in the dendrites can be revealed by confocal microscopy and the length and area were analyzed by Metamorph software. Figure 3A shows mitochondrial images of individual neurons in control and treated with 30 μM glutamate and 3 μM glycine glutamate for different times. Our data show that mitochondrial fragmentation occurred after neurons were exposed to glutamate. Frequency distribution analysis shows that glutamate stimulation reduced mitochondrial length and area as compared with neurons in control conditions (Figure 3B, C, Figure S1). The average length and area of mitochondria were progressively decreased with the treatment time (Figure 3D). However, the density of the mitochondria along the dendrites did not change with glutamate treatment (Figure 3D). These results demonstrate that glutamate induces neuronal mitochondrial fragmentation in a time-dependent manner.

**Figure 4:**
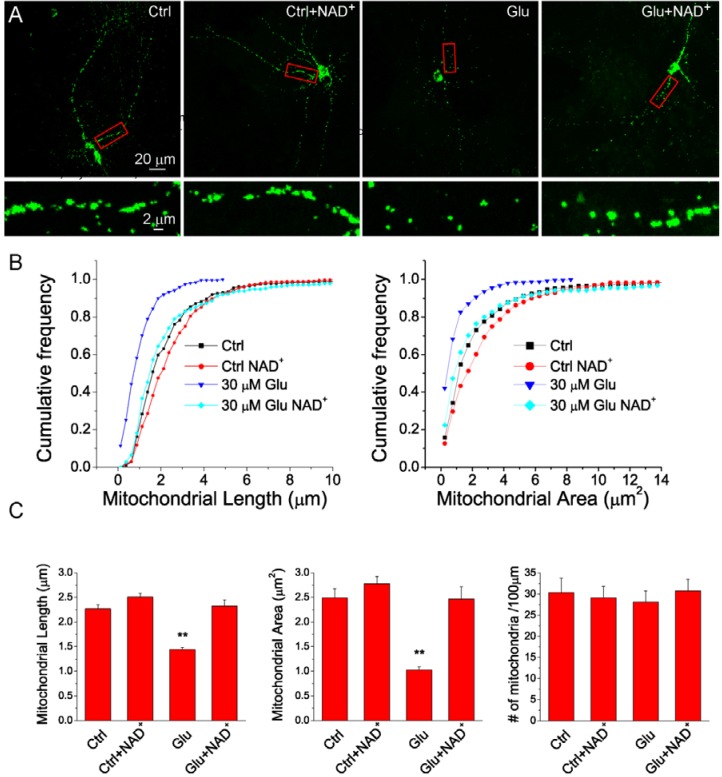
NAD^+^ inhibits glutamate-induced mitochondrial fragmentation. (**A**) Maximal projection confocal images of primary cortical neuron transfected with mito-AcGFP1. Neurons were treated without or with 30 μM glutamate together with 3 μM glycine for 24 h in the absence and presence of 15 mM NAD^+^. The lower panels are the high resolution images of the boxed region in upper panels; (**B**) Cumulative distribution curves of mitochondrial length and area under different conditions. The value of histogram interval (bin) is 0.5 μm^2^. Notice that glutamate treatment caused significant increase in the number of small mitochondria and this effect was attenuated by NAD^+^ supplement; (**C**) The summary of average mitochondrial length, area and density for each condition. Notice the density of mitochondria in neuronal dendrites was not affected by exogenous NAD^+^. Data were collected from 10–15 neurons in each condition and a total of 1941 mitochondria were measured. Results are shown as mean ± SE; ** *p* < 0.01 *versus* Ctrl, Ctrl + NAD^+^, and Glu + NAD^+^. ANOVA test.

As intact mitochondrial morphology plays an essential role in preserving mitochondrial normal function and promotes the neuronal survival after glutamate-induced excitotoxicity [26], we next tested whether NAD^+^ can prevent the glutamate-induced mitochondrial fragmentation. Neurons were treated with 30 μM glutamate and 3 μM glycine for 24 h in the presence or absence of 15 mM NAD^+^ and the mean mitochondrial length, area and density were analyzed as in Figure 3. Figure 4A shows mitochondrial images of individual neurons under different conditions. Frequency distribution analysis shows that NAD^+^ prevented glutamate-induced shortening of length and diminishment of area of mitochondria but NAD^+^ did not affect mitochondrial length and area under normal conditions (Figure 4B,C and Figure S2). However, the density of mitochondria in dendrites was not affected by NAD^+^ (Figure 4C). In summary, these data indicated that exogenous NAD^+^ can prevent glutamate-induced mitochondrial fragmentation.

### 2.4. NAD^+^ Restores Mitochondrial Biogenesis after Glutamate Stimulation

**Figure 5:**
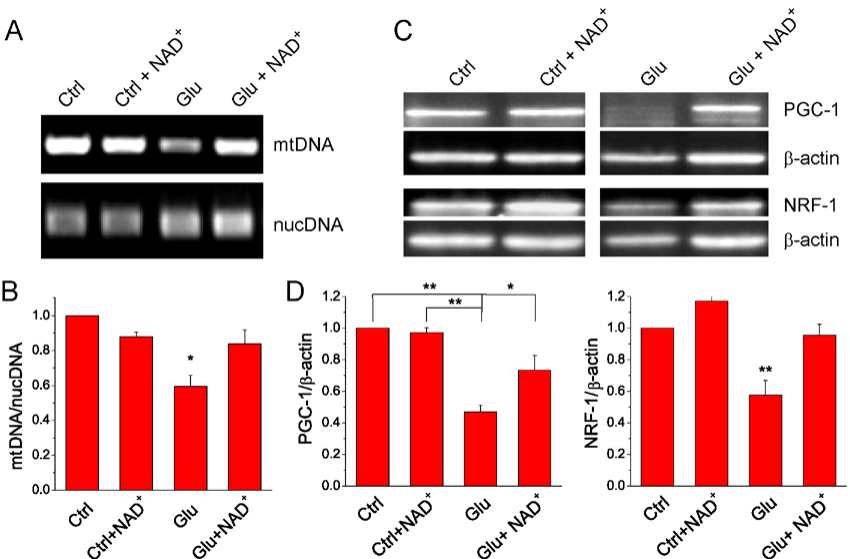
The effect of NAD^+^ on neuronal mitochondrial biogenesis after glutamate stimulation. (**A**) Agarose gel image of mtDNA and nucDNA. mtDNA and nucDNA were extracted from neurons cultured under different conditions and amplified by qPCR following. Neurons were treated with 30 μM glutamate together with 3 μM glycine for 24 h in the presence or absence of 15 mM NAD^+^; (**B**) Quantification of mtDNA amount in each condition. The data was presented as the ratio of mtDNA/nucDNA and normalized to the control condition using nucDNA as internal control. Notice that NAD^+^ prevented glutamate induced decrease of mtDNA content; (**C**) Western blot images for PGC-1 and NRF-1 in control and glutamate stimulated primary neuronal cultures in the presence and absence of 15 mM NAD^+^; (**D**) Quantitative analysis of PGC-1 and NRF-1 expression levels. The data were presented as the ratio of the proteins to β-actin and normalized to the control condition. All data (**B**,**D**) are shown as mean ± SE from three to six independent experiments. * *p* < 0.05, ** *p* < 0.01, *versus* Ctrl, Ctrl + NAD^+^ and Glu + NAD^+^, ANOVA test.

Experimental evidence suggests that improved mitochondrial biogenesis can reduce neuronal damage during cerebral ischemia through stimulating the renewal of functional mitochondria [27]. Quantity of mtDNA is an indicator of mitochondria content. Under ischemic conditions, permanent loss of mtDNA is suggestive of the failure of the mitochondrial renewal mechanisms, and thus leads to apoptotic neuronal death [27,28]. Hence we examined whether exogenous NAD^+^ can reduce mtDNA loss after glutamate excitotoxicity. We measured the amount of mtDNA and nuclear DNA (nucDNA) using qPCR to study the effect of NAD^+^ on mitochondrial content. Our results show that glutamate stimulation reduced the amount of mtDNA while NAD^+^ significantly attenuated the reduction of mtDNA (Figure 5A,B).

PGC-1 is essential for the regulation of mitochondrial biogenesis via accelerating the transcription of NRF-1 and mitochondrial transcription factor A (Tfam), and enhances mitochondrial proliferation [29]. To gain additional evidence for NAD^+^ protective effect on the mitochondrial biogenesis, the expression levels of PGC-1 and NRF-1 were investigated using Western blot analysis. Protein levels of PGC-1 and NRF-1 were significantly decreased after 24 h glutamate treatment. Supplementation of NAD^+^ to neuronal cultures significantly suppressed the reduction of the expression levels of both proteins (Figure 5C,D). Thus our results suggest that NAD^+^ can effectively suppress the impairment of glutamate-induced mitochondrial biogenesis through facilitating mtDNA amplification and PGC-1 and NRF-1 expression.

**Figure 6:**
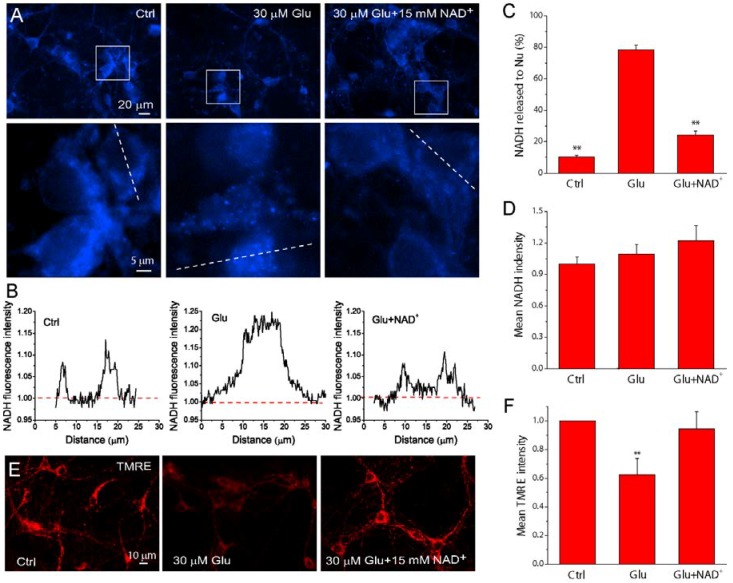
NAD^+^ preserves mitochondrial functional integrity after glutamate excitotoxicity. (**A**) Representative images of NADH auto-fluorescence under different conditions using fluorescence microscopy. The lower panels are the high resolution images of the boxed region in upper panels; (**B**) Line-scan of NADPH fluorescence from neurons indicated in (**A**), showing the localization of NADPH under different condition. The fluorescence was normalized to the background. Notice from (**A**,**B**) that glutamate stimulation caused NADH release from mitochondria and the concentration of NADPH in the nuclei; (**C**,**D**) The percentage of neurons with NADH localized in nuclei (**C**) and the average fluorescence intensity normalized with cell number in the field (**D**) under different conditions; (**E**,**F**) TMRE images of neurons **(E**) and the average fluorescence intensity normalized with cell number (**F**) with different treatments. The reduction of TMRE fluorescence indicated the loss of MMP. Results are shown as mean ± SE. For each condition, 5–8 fields were randomly chosen from 2 coverslips in 3–5 independent experiments. Primary mouse cortical neurons were stimulated by 30 μM glutamate together with 3 μM glycine for 24 h in the presence and absence of 15 mM NAD^+^. ** *p* < 0.01 *versus* Ctrl and Glu + NAD^+^, ANOVA test.

### 2.5. NAD^+^ Prevents the Loss of Mitochondrial Integrity and Function after Glutamate Stimulation

NADH is the reduced form of NAD^+^ and is an essential coenzyme for the oxidative enzymatic reactions in mitochondria. To further investigate the effect of NAD^+^ on mitochondrial functional integrity, cellular levels of NADH were measured by its auto-fluorescence using fluorescence microscopy in living primary neurons. NADH was mainly found inside mitochondria, and devoid in the nuclei in normal conditions but became diffusive in entire neurons after 24 h glutamate stimulation (Figure 6A,C), presumably due to the open of mitochondrial permeability transition pore (MPTP). However, supplementation of NAD^+^ to neurons significantly inhibited the release of NADH from mitochondria induced by glutamate excitotoxicity (Figure 6A,C). Line-scan analysis of NADH distributions further shows NADH is concentrated in mitochondria in normal conditions, but glutamate stimulation causes redistribution of NADH in the entire neurons and NAD^+^ could reverse the distribution (Figure 6B). We also measured the average NADH fluorescence intensity in the image field in each condition and normalized it to the number of neurons in the field. The results show that glutamate treatment did not alter the average cellular NADH levels compared with the control group regardless of the presence or the absence of NAD^+^ (Figure 6D). The influence of NAD^+^ on MMP was also monitored using the potentiometric fluorescent indicator tetramethylrhodamine ethyl ester (TMRE). The decrease of TMRE intensity after glutamate stimulation was significantly prevented by NAD^+^ (Figure 6E,F), suggesting the preservation of mitochondrial potential by NAD^+^ treatment. We therefore concluded that NAD^+^ can preserve mitochondrial functional integrity during glutamate stimulation.

## 3. Discussion

NAD^+^ is an essential cofactor for cellular metabolism and energy production. Therefore, maintenance of NAD^+^ levels is critical for preserving neuronal bioenergetics and promoting cell survival after cerebral ischemia. In the present study, we used glutamate excitotoxicity as *in vitro* ischemic model to mimic the penumbra in focal ischemic stroke and studied the effect of NAD^+^ on neuronal apoptosis and the mitochondrial dysfunction in cultured neurons. Our main findings of this study include the following: (1) NAD^+^ reduced neuronal apoptotic death and prevented AIF translocation after glutamate stimulation; (2) NAD^+^ inhibited glutamate-induced mitochondrial fragmentation; (3) NAD^+^ suppressed glutamate-induced impairment of mitochondrial biogenesis; (4) Supplementation of NAD^+^ during glutamate treatment attenuated the loss of mitochondrial mass, reduction of MMP, and blocked NADH release presumably through the opening of MPTP. These results indicate that NAD^+^ exerts its neuroprotective effect after glutamate excitotoxicity through improving mitochondrial biogenesis and promote mitochondrial function.

Previous studies in our and other groups have shown that supplement of NAD^+^ has a neuronal protective effect in *in vitro* and *in vivo* ischemic models and maintaining intracellular NAD^+^ levels is important in promoting cell survival during ischemia [12,13,14]. Although the effect of NAD^+^ on apoptosis induced by chemical apoptotic triggers including rotenone and *N*-methyl-*N'*-nitro-*N*-nitrosoguanidine (MNNG) were studied on non-neuronal cell lines such as PC12, Hela, Raw and HepG2 cells [30,31], the present study further shows that exogenous NAD^+^ can reduce apoptotic neuronal death in glutamate excitotoxicity, a more relevant model to ischemic stroke. Poly (ADP-ribose) polymerase-1 (PARP-1), an NAD^+^ consuming enzyme, is overactivated in ischemic stroke and has been shown to play a dominant role in neuronal death after ischemic stroke [32,33]. Studies from MNNG-triggered neuronal injury model also showed that MNNG-induced PARP-1 activation triggers AIF translocation, a prominent phenomenon of apoptosis [17,34,35]; however, supplementation of NAD^+^ increases PARP-1 activity suggesting the neuroprotective effect of NAD^+^ in this model is not due to PARP-1 activation and NAD^+^ depletion is upstream of mitochondrial AIF translocation. Here, we also show that supplement of NAD^+^ reduced translocation of AIF from mitochondria to nuclei, thus prevention of AIF translocation by NAD^+^ supplement after glutamate excitotoxicity might act through preserving high intracellular NAD^+^ rather than activation of PARP-1 *per se*. On the other hand, it was reported that ischemia causes the reduction of NAD^+^ levels in ischemic models of mouse and cultured neurons [13,15,16], and inhibition of AIF translocation protected the neurons against apoptosis in hippocampal CA1 region after transient forebrain ischemia [36]. These findings suggest that preserving cellular NAD^+^ level is critical for cell survival during *in vitro* and *in vivo* ischemia and such protective effects might exert through preventing the release of AIF from mitochondria to nucleus. Since AIF is one of the major factors in the mitochondrial pathway that is responsible for the apoptotic neuronal death, our findings suggest that NAD^+^ protects neuronal apoptosis against glutamate excitotoxicity through the prevention of AIF translocation, thus inhibiting mitochondrial failure during glutamate stimulation. Since it has been known that extracellular NAD^+^ can enter different types of cell including primary cultured neurons [31,34] and increase more mitochondrial NAD^+^ content than nucleocytosolic content [31], the effect of extracellular NAD^+^ on AIF translocation and apoptosis might be through the increase of mitochondrial NAD^+^ levels. We did not study whether NAD^+^ reduces caspase-3-dependent apoptosis, however, other studies reported that glutamate excitotoxicity-induced apoptosis could be mediated by both caspase-3-dependent pathway with mitochondrial cytochrome C release and caspase-independent pathway involving AIF translocation in primary cortical neurons [37]. However, it was reported that extracellular NAD^+^ could suppress mefloquine induced caspases-3-mediated apoptosis in the spiral ganglion neurons of cultured cochlear organs [38], raising the possibility that extracellular NAD^+^ might also regulate glutamate excitotoxicity through a caspase-dependent apoptotic pathway.

Although NAD^+^ has the ability to prevent glutamate-induced apoptosis, the effect of NAD^+^ on mitochondrial biogenesis and function in glutamate excitotoxicity has not been well studied. Alterations in mitochondrial morphology have been associated with both necrotic and apoptotic neuronal death and reservation of mitochondrial morphology can protect neurons against NMDA-induced excitotoxicity injury [25,26]. Accordingly, we further studied the effect of NAD^+^ on the glutamate-induced alteration of mitochondrial morphology. We measured and compared average mitochondrial length and area before and after glutamate treatment. Our results show that the degree of mitochondrial fragmentation manifested by decrease in length and area is increased progressively over time after glutamate stimulation, which is consistent with result from a previous report [39]. Our results further showed that supplementation of NAD^+^ inhibited the glutamate-mediated mitochondrial fragmentation. These results indicate a role of NAD^+^ in the prevention of structural collapse of mitochondria in response to glutamate excitotoxicity. Mitochondrial morphology is dynamic and can be regulated via fusion and fission [40]. Abnormal mitochondrial dynamics after glutamate stimulation described in this study may be a consequence of the changes in the expression of mitochondrial fission and fusion proteins such as dynamin-related protein-1 (DRP-1) and mitofusin-1 (mfn-1) respectively [25,41,42]. Therefore, it will be interesting to further explore whether DRP-1 and mfn-1 play a role in maintaining mitochondrial structural integrity during glutamate stimulation in the presence of NAD^+^ treatment. Interestingly, we found that mitochondrial density along neuronal process has not been affected by the glutamate stimulation in the presence or absence of NAD^+^, but supplementation of NAD^+^ restore the average area of mitochondria. This suggests that glutamate stimulation may not break the balance between mitochondrial fusion and fission, but only affect the overall mitochondrial mass and biogenesis while NAD^+^ can prevent the loss of mitochondrial mass, but does not affect mitochondrial number. As increase in mitochondrial mass is also an indication of increased mitochondrial biogenesis [43], thus NAD^+^ can improve mitochondrial biogenesis after glutamate excitotoxicity.

Healthy neurons generate new functional mitochondria through coordinated action of transcriptional factors and co-activators. Mitochondrial biogenesis is highly correlated with NAD^+^ levels [9,10,11,44], but mitochondrial biogenesis is impaired in ischemic neurons [27,28]. To further investigate the mechanism of NAD^+^ protective effect on neurons during glutamate stimulation, we examined the effect of NAD^+^ supplementation on the mitochondrial biogenesis, a critical event that determines cell survival during glutamate stimulation. Our results show that the relative amount of mtDNA to nucDNA in primary cortical neurons after 24 h glutamate stimulation was decreased, but NAD^+^ attenuated the decrease. These results are in agreement with a previous report demonstrating that prevention of mtDNA content decrease restored mitochondrial biogenesis and reduced infarct size in a mouse permanent middle cerebral artery occlusion (pMCAO) model [27]. Mitochondrial biogenesis is also determined by key regulators such as PGC-1 and NRF-1 [9,27,45]. It has been reported that up-regulation of these two proteins protects the neurons against OGD injury in neurons [45]. We found that PGC-1 and NRF-1 were decreased after glutamate excitotoxicity but NAD^+^ restored their levels. These results indicate that NAD^+^ can either inhibit mitochondrial loss or facilitate mitochondrial proliferation during glutamate stimulation.

Beyond its well-established role in cellular energy metabolism, intracellular NAD^+^ level has been shown to affect the enzymatic activity of various NAD^+^-dependent enzymes that involved in cell survival. One of those enzymes, Sirt-1, may also contribute to the protective effect of NAD^+^ on the regulation of mitochondrial biogenesis after glutamate stimulation. The expression of Sirt-1 was regulated by energy availability in hypothalamus, suggesting a potential role for Sirt-1 in regulating energy balance in the CNS [46]. Activation of SIRT1 also has a beneficial effect on mitochondrial biogenesis [9,10,11,44]. Specially, increasing Sirt-1 expression can downstream activate the expression of PGC-1 and then stimulate mitochondrial biogenesis [9,47]. Since the cellular functions of Sirt-1 are dependent upon its deacetylase activity which requires NAD^+^, increase in mitochondrial biogenesis by NAD^+^ supplementation may act through the activation of Sirt-1. Nonetheless, whether the effect of NAD^+^ on the up-regulation of PGC-1 and NRF-1 expression after glutamate excitotoxicity reported in this study depends on Sirt-1 activity is unknown, and will be worthwhile to explore in future studies.

NADH is a derivative compound of NAD^+^. Maintenance of NAD^+^/NADH ratio is critical for mitochondrial function such as ATP synthesis in TCA cycle and oxidative phosphorylation in mitochondria. Neurons have high metabolic activities and are especially sensitive to the ischemia-induced energy failure. Since enzymatic reactions related to ATP synthesis are dependent on NADH as the source of reducing power, the decrease of NADH level in the mitochondria will exacerbate ischemia-induced energy stress. The location and content of NADH can be directly estimated by its auto-fluorescence using fluorescence microscopy. NADH is located mainly in mitochondria under normal conditions, however, although the overall NADH auto-fluorescence intensity did not significantly change, NADH was more concentrated in nuclei after glutamate stimulation, suggesting glutamate-induced release of mitochondrial NADH presumably through the opening of MPTP. In the presence of NAD^+^, the release of NADH from mitochondria is inhibited during glutamate stimulation. Moreover, our results also demonstrate that NAD^+^ prevents the glutamate induced depolarization of MMP. Thus, our results suggest that extracellular supplementation of NAD^+^ could maintain mitochondrial integrity after glutamate excitotoxicity. This notion is supported by recent studies demonstrating that prevention of prolonged MPTP opening reduced the ethanol-induced decrease in MMP depolarization and release of NADH in mouse neuronal cultures [48]. Mitochondrial impairment resulting from prolonged opening of the MPTP has been shown to play a significant role in perturbation of cellular bioenergetics and therefore factors that can inhibit the opening of MPTP can prevent the mitochondrial dysfunction induced by energy stress after glutamate excitotoxicity. Thus our results suggest NAD^+^ can maintain mitochondrial integrity during glutamate excitotoxicity through enhanced energy metabolism.

Different mechanisms by which NAD^+^ protects neuronal death have been suggested. It was reported that NAD^+^ can protect neurons in OGD model through restoration of DNA repair [12]. Another study showed that NAD^+^ can block autophagy in MCAO model [14]. Our previous study suggested that NAD^+^ can inhibit the impairment of mitochondrial biogenesis, but detailed study has not been done [13]. Our current study established that NAD^+^ can ameliorate apoptosis after glutamate excitotoxicity through preserving mitochondrial biogenesis and functional integrity. Future studies are required to evaluate the beneficial effect of NAD^+^ on mitochondrial function and biogenesis using *in vivo* ischemic stroke model.

## 4. Materials and Methods

### 4.1. Primary Neuronal Cultures and in Vitro Ischemia Model

Primary mouse cortical neuronal cultures were prepared from embryonic day 15/16 (E15/16) C57BL/6J mice as described in our previous study [13]. The isolated cells were plated onto poly-l-lysine-coated (100 μg/mL) (Cat. No. P9155; Sigma-Aldrich, St. Louis, MO, USA) tissue culture plates or glass coverslips of 12 mm in diameter (Cat. No.1943-10012; Fisher Scientific, Waltham, MA, USA) in a 24-well culture plate with Dulbecco’s modified Eagle medium/nutrient F12 (Cat. No. 11320-033; Invitrogen) supplemented with 10% heated-inactivated fetal bovine serum (Cat. No. S11150; Atlanta Biological, Norcross, GA, USA) for 5 h. The medium was then changed to Neurobasal Media (Cat. No. 21103-049; Invitrogen) containing 2% B27 serum free supplements (Cat. No. 17504-044, Invitrogen), 0.5 mM l-glutamine (Cat. No. 25030; Invitrogen) and 50 U/ml Penicillin/Streptomycin (Cat. No. 15140; Invitrogen). In order to suppress the growth of glial cells and increase the purity of neuronal cultures, 5 μM Cytosine β-D-arabino-furanoside (Cat. No. C1768; Sigma-Aldrich) was added 24 h after plating. The cultures were maintained in an incubator at 37 °C with a humidified atmosphere of 5% CO_2_ and 95% air. Cultures were maintained with 50% medium changes in every 3 days. Experiments were conducted 9–11 days *in vitro* (DIV) as the neurons were matured at this age and vulnerable to glutamate-induced excitotoxicity. To mimic ischemia-like conditions *in vitro*, primary neuronal cultures were exposed to 30 μM glutamate with 3 μM glycine for 24 h or 100 μM glutamate with 10 μM glycine for 3 h.

### 4.2. Immunofluorescent Staining

Neurons seeded on the coated-glass coverslips were washed with 1× PBS three times and fixed for 15 min with ice-cold 4% paraformaldehyde (PFA) in 1× PBS and then rinsed three times with 1× PBS carefully. Cells were then permeabilized with ice-cold 0.1% Triton X-100 in 1× PBS for 10 min and blocked with 5% serum in 1× PBS at room temperature for 1 h. Neurons were incubated with rabbit anti-AIF polyclonal antibody (1:200; Cat. No. PRS2301; Sigma-Aldrich) in 1% serum, 0.1% Triton X-100 in 1× PBS at 4 °C overnight. The cells were washed three times each for 5 min with 1× PBS and then incubated with Rhodamine-conjugated donkey anti-rabbit IgG (1:200; Cat. No. AP182R; Millipore, Billerica, MA, USA) in 1% serum, 0.1% Triton X-100 in 1× PBS for 1 h in the dark at room temperature. The nuclei were stained with DAPI. The samples were subjected to fluorescence detection using a Nikon FN1 epi-fluorescence microscopy equipped with a CoolSNAP-EZ CCD camera (Photometrics, Tucson, AZ, USA) or an Olympus confocal microscope and analyzed by MetaMorph software (Molecular Devices, Sunnyvale, CA, USA). Line-scan analysis of AIF fluorescence was done by MetaMorph software. Data were averaged from three independent experiments for each condition, with 2–3 glass cover slips and 6–8 fields per glass cover slips in each experiment.

### 4.3. Apoptotic Neuronal Death Assay

We used TUNEL and Hoechst 33342 staining to identify apoptotic neuronal death after glutamate stimulation. Briefly, for TUNEL staining, cultured neurons were fixed with 4% PFA in PBS for 30 min and incubated with permeabilisation solution (0.1% sodium citrate in 0.1% Triton X-100) for 2 min on ice, and subsequently with TUNEL reaction mixture (*In Situ* Cell Death Detection Kit, Fluorescein; Cat. No. 11684795910; Roche, Basel, Switzerland) in a humidified environment for 1 h at 37 °C in the dark. Samples were directly analyzed under a Nikon FN1 epi-fluorescence microscopy with a 40× magnification objective. The total number of neurons was counted based on DAPI stained nuclei and the number of apoptotic neurons was counted based on TUNEL+ cells. Apoptotic cell death was also quantifies by counting condensed nuclei stained with Hoechst 33342 (5 μg/mL) staining for 30 min in the dark. The counting was performed by an experimenter blind to experimental conditions. The neuronal survival rate was defined as the ratio of the number of TUNEL-cells to the total number of neurons determined by DAPI-stained nuclei.

### 4.4. Measurement of Mitochondrial Morphology

Neurons were first transfected with mito-AcGFP using lipofectamine 2000 (Cat. No.11668027; Invitrogen). To study the effect of NAD^+^ on the mitochondrial fragmentation after glutamate excitotoxicity, neuronal cultures were treated with 30 μM glutamate and 3 μM glycine for 24 h in the presence or absence of 15 mM NAD^+^. Neurons were then fixed in 4% PFA and imaged with a FluoView 1000 Olympus confocal microscope with a 60× oil immersion objective. Mitochondrial length and area were calculated using MetaMorph offline software. All the images were adjusted to a same intensity scale. User-defined thresholds for pixel intensity and object size were used to identify mitochondrial area, length and density. For each neuron, mitochondria located within 100 μm in dendrite from soma were selected for analysis [43].

### 4.5. Assays of Mitochondrial Membrane Potential (MMP) and NADH Distribution

MMP were evaluated by labeling living neurons with TMRE (Cat. No. T-688; Invitrogen) respectively similar to methods described previously [13,15]. Neurons cultured on glass coverslips were loaded with ACSF containing 25 nM TMRE for 20 min at room temperature. The cells were also double stained with Hoechst 33342 to determine the cell density. TMRE fluorescence and NADH auto-fluorescence were detected with a Nikon FN1 epi-fluorescence microscopy equipped with a CoolSNAP-EZ CCD camera. The fluorescence intensities of TMRE and NADH, and line-scan of NADH distribution were analyzed by MetaMorph offline software. The mean fluorescence intensities of TMRE and NADH were defined as the ratio of average TMRE and NADH fluorescence intensity in entire fields to the number of cells. The percentage of neurons with release of NADH from mitochondria was defined as the ratio of the number of neurons with concentrated NADH nuclei to the total number of neurons [48].

### 4.6. mtDNA Quantification

qPCR was used to quantify the relative abundance of intact mtDNA [13,27,49]. The conditions of PCR reaction and the sequence of primers were described in our previous study [13]. Total DNA of cultured neurons was extracted and purified using the genomic DNA extraction kit (Cat. No. 69504; Qiagen, Venlo, Netherlands). The total DNA derived from neurons in one well from 12-well plates with equal amount was added to the polymerase chain reaction (PCR) mixture with GoTaq Flexi DNA Polymerase (Cat. No. PRM8295; Promega, Fitchburg, WI, USA). PCR products are 655 bp for nucDNA and 464 bp for mtDNA. DNA samples were separated on a 1% agarose gel and stained by Ethidium Bromide (0.5 mg/mL). The images were acquired by a Fuji LAS 3000 densitometer. The density of the band was calculated using Image J software. The ratio of mtDNA content to nucDNA content was calculated.

### 4.7. Western Blot Analysis

Western blot was used to analyze PGC-1 and NRF-1 expression levels in neuronal cultures without and with treatment of 30 μM glutamate plus 3 μM glycine in the presence or absence of 15 mM NAD^+^ for 24 h. Protein samples were separated by 10% SDS-polyacrylamide gel electrophoresis and transferred to PVDF membranes. The membranes were blocked by 5% non-fat dry milk and incubated in a rabbit antibody against PGC-1 (1:1000; Cat. No. sc-13067; Santa Cruz Biotechnology, Dallas, TX, USA) or NRF-1 (1:1000; Cat. No. sc-33771; Santa Cruz Biotechnology) and HRP-conjugated second antibody. Finally, the protein bands were visualized by ECL Western blotting detection system. β-actin was used as a control for equal loading of the total protein.

### 4.8. Statistical Analysis

Data are expressed as means ± SEM obtained from at least three independent experiments using cortical neurons cultured from different mice. Statistical comparisons were made by two-sample *t*-test or a one-way ANOVA followed by Tukey test was used for multiple groups and *p*-value <0.05 and <0.01 was considered to be significant and indicated as one and two stars respectively.

## 5. Conclusions

In summary, our study revealed that NAD^+^ can ameliorate apoptotic neuronal death, inhibit AIF translocation, reduce the impairment of mitochondrial biogenesis, prevent the loss of mitochondria mass, and suppress MMP depolarization and release of NADH after glutamate excitotoxicity. Therefore, mitochondria-dependent apoptotic signal pathway contributes to, at least in part, the mechanisms underlying NAD^+^ protective effect during glutamate-induced apoptosis. Our results suggest that maintaining cellular energy homeostasis, particularly NAD^+^ levels, is a valuable neuroprotective strategy for ischemic stroke. Our results also provide insights into other therapeutic avenues related to mitochondrial biogenesis.

## Supplementary Materials

Supplementary figures can be found at http://www.mdpi.com/1422-0067/15/11/20449/s1.
